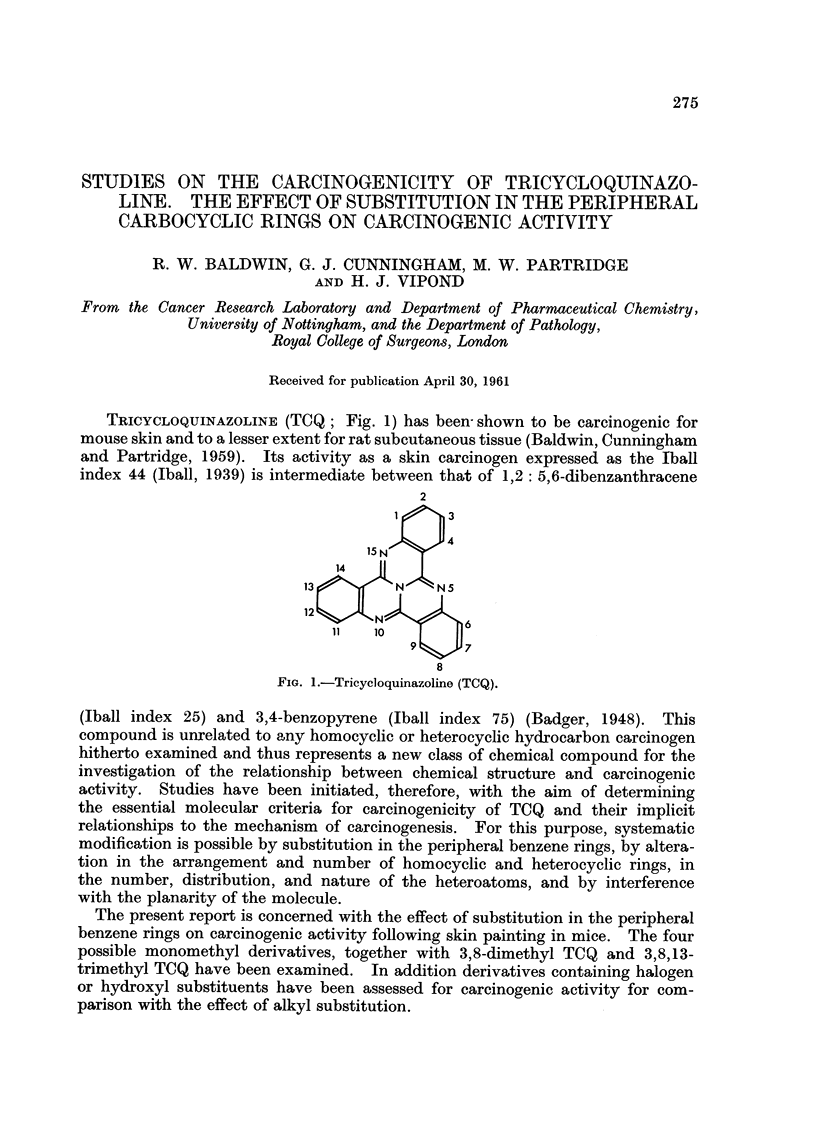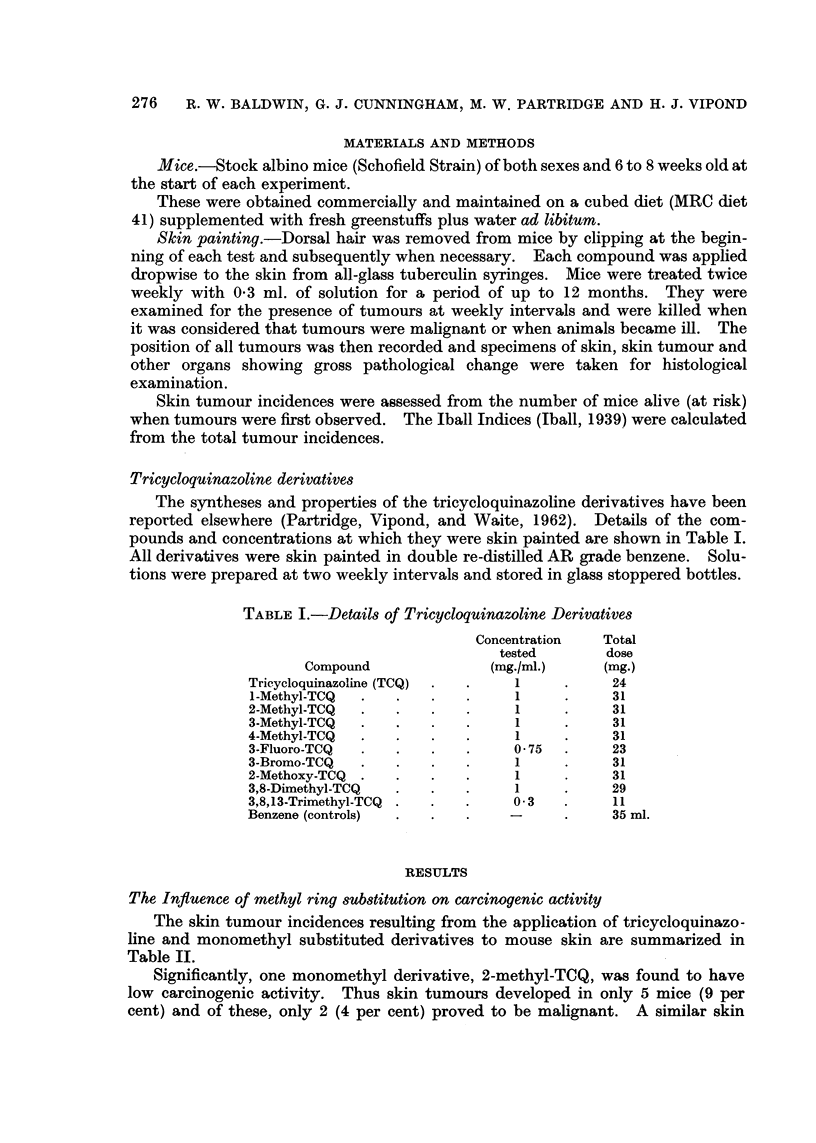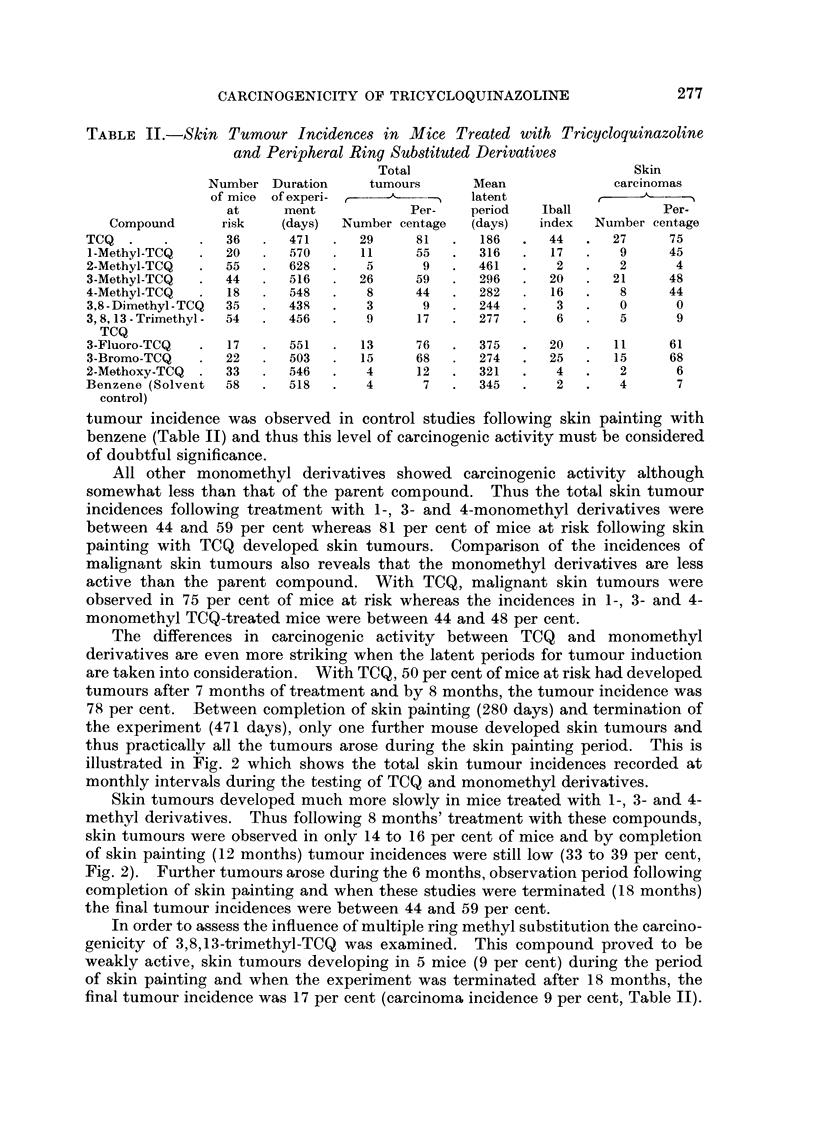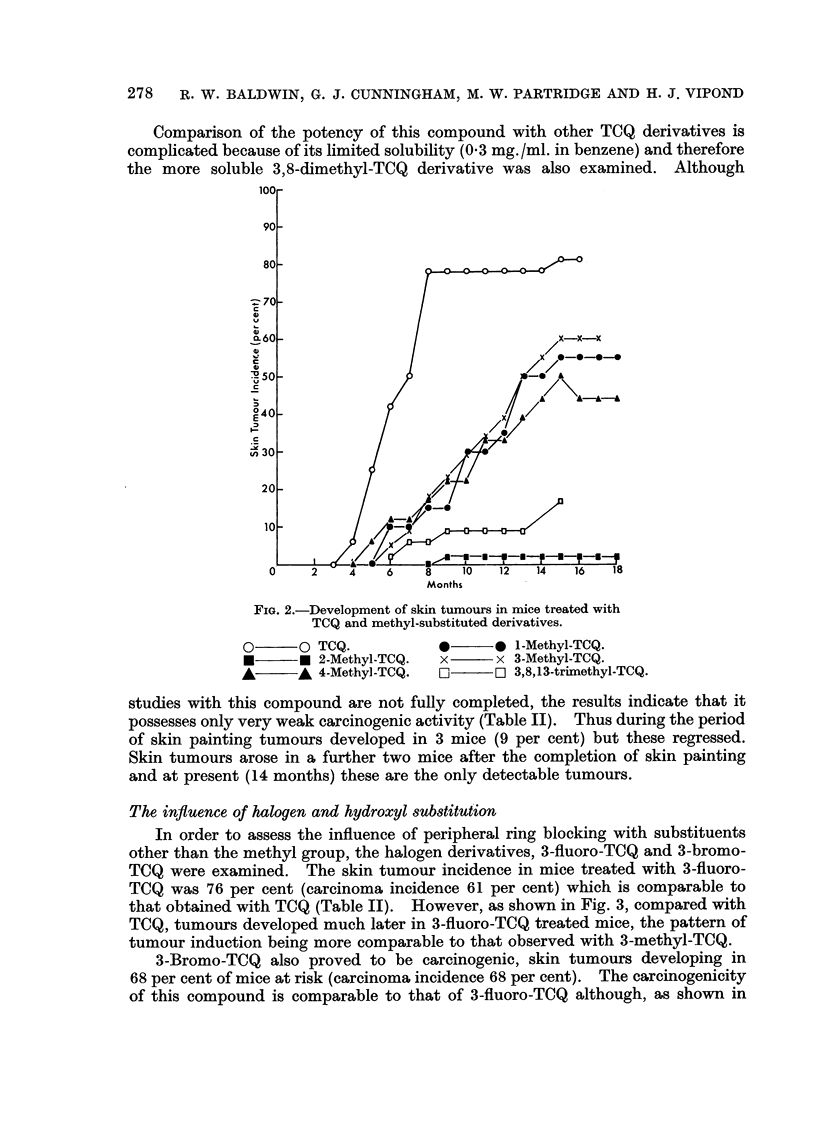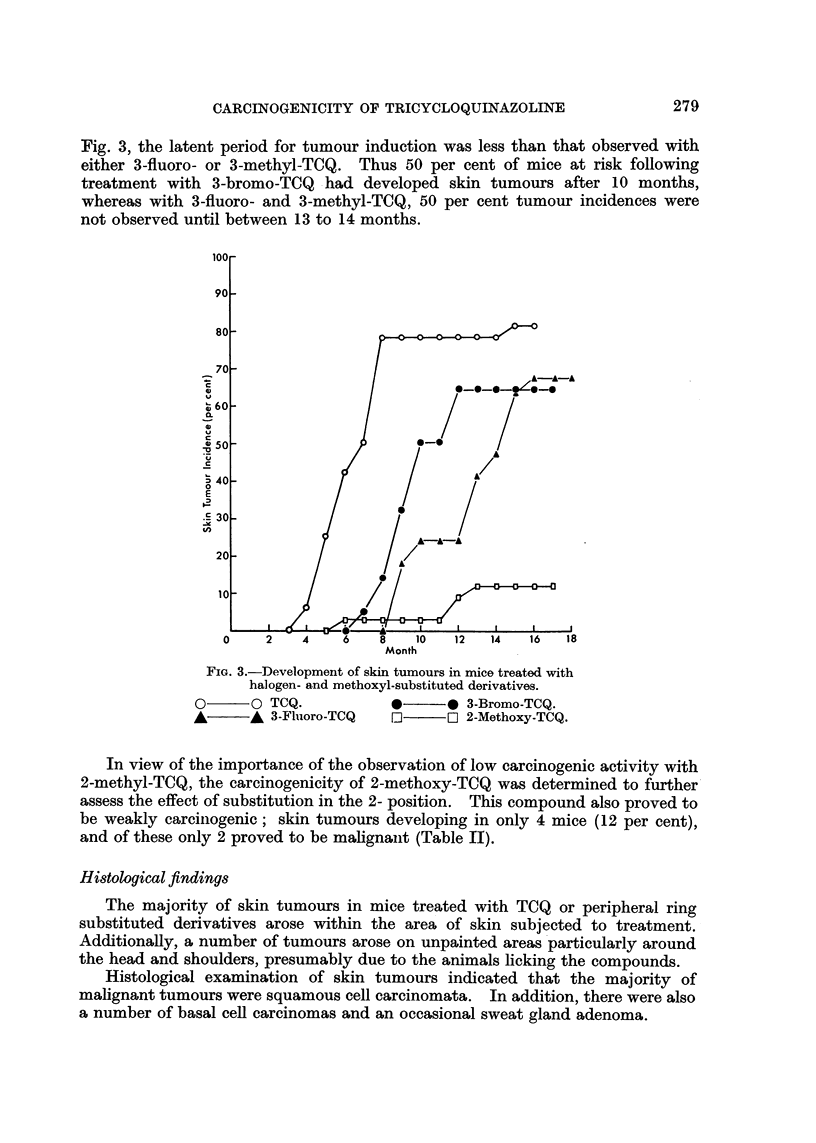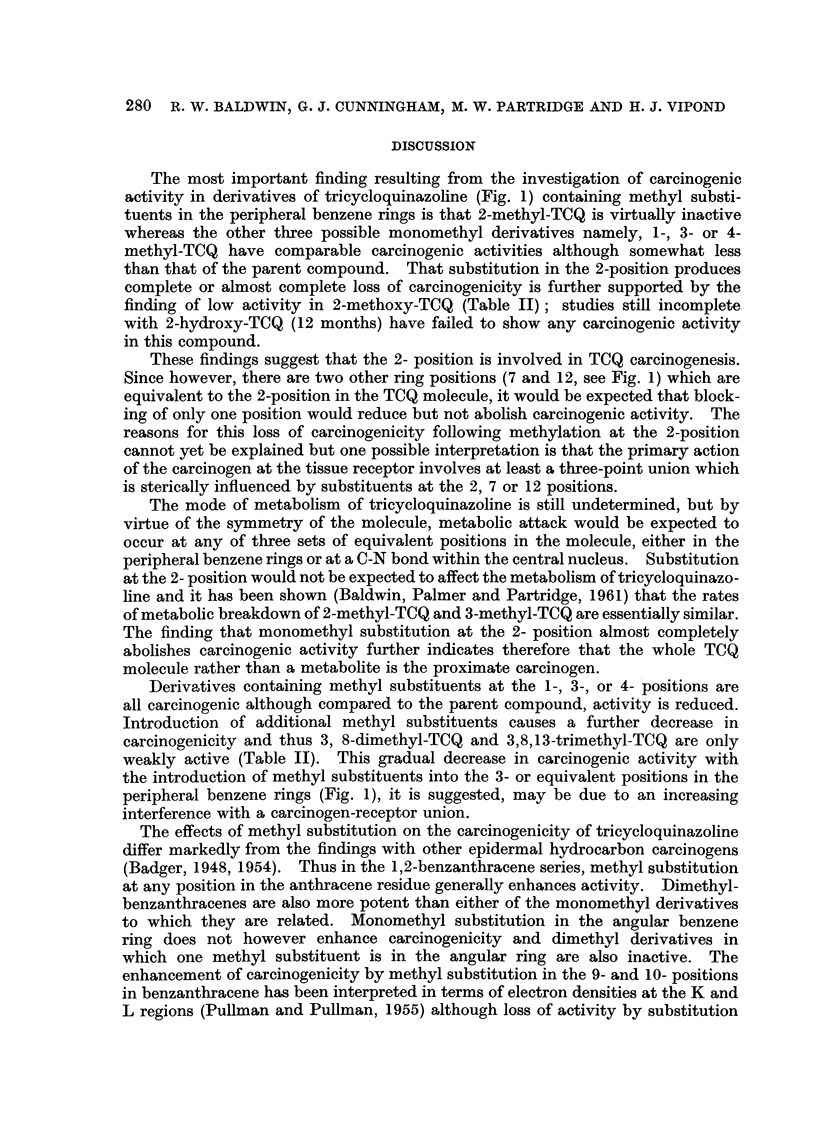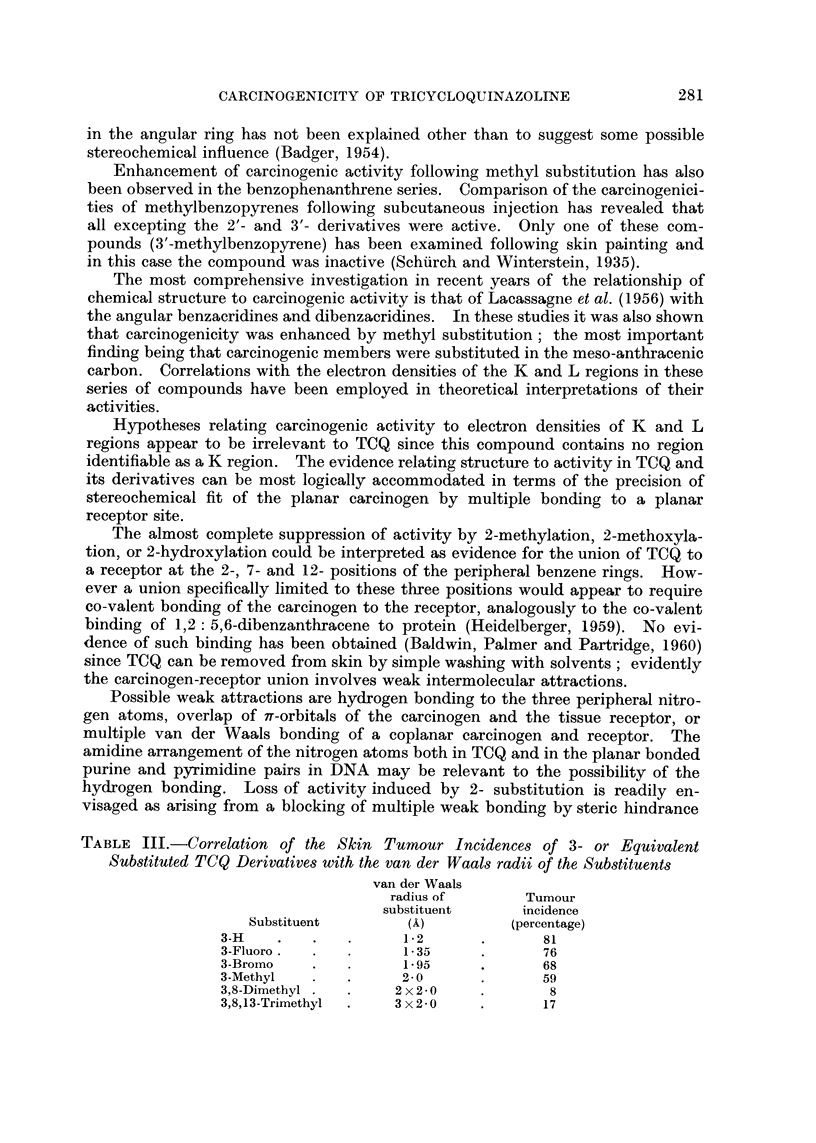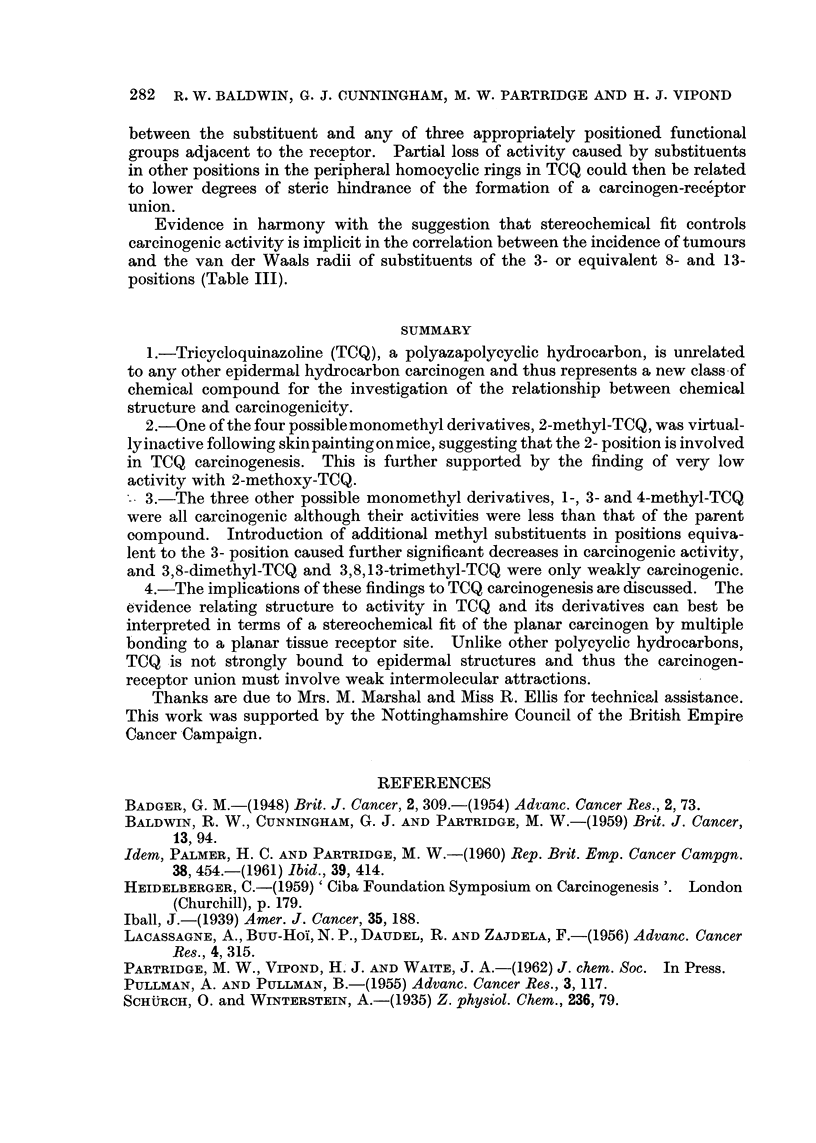# Studies on the Carcinogenicity of Tricycloquinazoline. The effect of Substitution in the Peripheral Carbocyclic Rings on Carcinogenic Activity

**DOI:** 10.1038/bjc.1962.32

**Published:** 1962-06

**Authors:** R. W. Baldwin, G. J. Cunningham, M. W. Partridge, H. J. Vipond


### STUDIES ON THE CARCINOGENICITY OF TRICYCLOQUINAZOLINE. THE EFFECT OF SUBSTITUTION IN THE PERIPHERAL CARBOCYCLIC RINGS ON CARCINOGENIC ACTIVITY

R. W. BALDWIN, G. J. CUNNINGHAM, M. W. PARTRIDGE AND H. J. VIPOND

*From the Cancer Research Laboratory and Department of Pharmaceutical Chemistry, University of Nottingham, and the Department of Pathology, Royal College of Surgeons, London*

Received for publication April 30, 1961

TRICYCLOQUINAZOLINE (TCQ ; Fig. 1) has been shown to be carcinogenic for mouse skin and to a lesser extent for rat subcutaneous tissue (Baldwin, Cunningham and Partridge, 1959). Its activity as a skin carcinogen expressed as the Iball index 44 (Iball, 1939) is intermediate between that of 1,2 : 5,6-dibenzanthracene

FIG. 1.—Tricycloquinazoline (TCQ).

(Iball index 25) and 3,4-benzopyrene (Iball index 75) (Badger, 1948). This compound is unrelated to any homocyclic or heterocyclic hydrocarbon carcinogen hitherto examined and thus represents a new class of chemical compound for the investigation of the relationship between chemical structure and carcinogenic activity. Studies have been initiated, therefore, with the aim of determining the essential molecular criteria for carcinogenicity of TCQ and their implicit relationships to the mechanism of carcinogenesis. For this purpose, systematic modification is possible by substitution in the peripheral benzene rings, by alteration in the arrangement and number of homocyclic and heterocyclic rings, in the number, distribution, and nature of the heteroatoms, and by interference with the planarity of the molecule.

The present report is concerned with the effect of substitution in the peripheral benzene rings on carcinogenic activity following skin painting in mice. The four possible monomethyl derivatives, together with 3,8-dimethyl TCQ and 3,8,13-trimethyl TCQ have been examined. In addition derivatives containing halogen or hydroxyl substituents have been assessed for carcinogenic activity for comparison with the effect of alkyl substitution.

### MATERIALS AND METHODS

*Mice.*—Stock albino mice (Schofield Strain) of both sexes and 6 to 8 weeks old at the start of each experiment.

These were obtained commercially and maintained on a cubed diet (MRC diet 41) supplemented with fresh greenstuffs plus water *ad libitum.*

*Skin painting.*—Dorsal hair was removed from mice by clipping at the beginning of each test and subsequently when necessary. Each compound was applied dropwise to the skin from all-glass tuberculin syringes. Mice were treated twice weekly with 0·3 ml. of solution for a period of up to 12 months. They were examined for the presence of tumours at weekly intervals and were killed when it was considered that tumours were malignant or when animals became ill. The position of all tumours was then recorded and specimens of skin, skin tumour and other organs showing gross pathological change were taken for histological examination.

Skin tumour incidences were assessed from the number of mice alive (at risk) when tumours were first observed. The Iball Indices (Iball, 1939) were calculated from the total tumour incidences.

### *Tricycloquinazoline derivatives*

The syntheses and properties of the tricycloquinazoline derivatives have been reported elsewhere (Partridge, Vipond, and Waite, 1962). Details of the compounds and concentrations at which they were skin painted are shown in Table I. All derivatives were skin painted in double re-distilled AR grade benzene. Solutions were prepared at two weekly intervals and stored in glass stoppered bottles.

TABLE I.—*Details of Tricycloquinazoline Derivatives*

| Compound | Concentration tested (mg./ml.) | Total dose (mg.) |
|---|---|---|
| Tricycloquinazoline (TCQ) | 1 | 24 |
| 1-Methyl-TCQ | 1 | 31 |
| 2-Methyl-TCQ | 1 | 31 |
| 3-Methyl-TCQ | 1 | 31 |
| 4-Methyl-TCQ | 1 | 31 |
| 3-Fluoro-TCQ | 0·75 | 23 |
| 3-Bromo-TCQ | 1 | 31 |
| 2-Methoxy-TCQ | 1 | 31 |
| 3,8-Dimethyl-TCQ | 1 | 29 |
| 3,8,13-Trimethyl-TCQ | 0·3 | 11 |
| Benzene (controls) | — | 35 ml. |

### RESULTS

### *The Influence of methyl ring substitution on carcinogenic activity*

The skin tumour incidences resulting from the application of tricycloquinazoline and monomethyl substituted derivatives to mouse skin are summarized in Table II.

Significantly, one monomethyl derivative, 2-methyl-TCQ, was found to have low carcinogenic activity. Thus skin tumours developed in only 5 mice (9 per cent) and of these, only 2 (4 per cent) proved to be malignant. A similar skin

TABLE II.—*Skin Tumour Incidences in Mice Treated with Tricycloquinazoline and Peripheral Ring Substituted Derivatives*

| Compound | Number of mice at risk | Duration of experiment (days) | Total tumours Number | Total tumours Percentage | Mean latent period (days) | Iball index | Skin carcinomas Number | Skin carcinomas Percentage |
|---|---|---|---|---|---|---|---|---|
| TCQ . . . | 36 | 471 | 29 | 81 | 186 | 44 | 27 | 75 |
| 1-Methyl-TCQ | 20 | 570 | 11 | 55 | 316 | 17 | 9 | 45 |
| 2-Methyl-TCQ | 55 | 628 | 5 | 9 | 461 | 2 | 2 | 4 |
| 3-Methyl-TCQ | 44 | 516 | 26 | 59 | 296 | 20 | 21 | 48 |
| 4-Methyl-TCQ | 18 | 548 | 8 | 44 | 282 | 16 | 8 | 44 |
| 3,8-Dimethyl-TCQ | 35 | 438 | 3 | 9 | 244 | 3 | 0 | 0 |
| 3, 8, 13-Trimethyl-TCQ | 54 | 456 | 9 | 17 | 277 | 6 | 5 | 9 |
| 3-Fluoro-TCQ | 17 | 551 | 13 | 76 | 375 | 20 | 11 | 61 |
| 3-Bromo-TCQ | 22 | 503 | 15 | 68 | 274 | 25 | 15 | 68 |
| 2-Methoxy-TCQ | 33 | 546 | 4 | 12 | 321 | 4 | 2 | 6 |
| Benzene (Solvent control) | 58 | 518 | 4 | 7 | 345 | 2 | 4 | 7 |

tumour incidence was observed in control studies following skin painting with benzene (Table II) and thus this level of carcinogenic activity must be considered of doubtful significance.

All other monomethyl derivatives showed carcinogenic activity although somewhat less than that of the parent compound. Thus the total skin tumour incidences following treatment with 1-, 3- and 4-monomethyl derivatives were between 44 and 59 per cent whereas 81 per cent of mice at risk following skin painting with TCQ developed skin tumours. Comparison of the incidences of malignant skin tumours also reveals that the monomethyl derivatives are less active than the parent compound. With TCQ, malignant skin tumours were observed in 75 per cent of mice at risk whereas the incidences in 1-, 3- and 4-monomethyl TCQ-treated mice were between 44 and 48 per cent.

The differences in carcinogenic activity between TCQ and monomethyl derivatives are even more striking when the latent periods for tumour induction are taken into consideration. With TCQ, 50 per cent of mice at risk had developed tumours after 7 months of treatment and by 8 months, the tumour incidence was 78 per cent. Between completion of skin painting (280 days) and termination of the experiment (471 days), only one further mouse developed skin tumours and thus practically all the tumours arose during the skin painting period. This is illustrated in Fig. 2 which shows the total skin tumour incidences recorded at monthly intervals during the testing of TCQ and monomethyl derivatives.

Skin tumours developed much more slowly in mice treated with 1-, 3- and 4-methyl derivatives. Thus following 8 months' treatment with these compounds, skin tumours were observed in only 14 to 16 per cent of mice and by completion of skin painting (12 months) tumour incidences were still low (33 to 39 per cent, Fig. 2). Further tumours arose during the 6 months, observation period following completion of skin painting and when these studies were terminated (18 months) the final tumour incidences were between 44 and 59 per cent.

In order to assess the influence of multiple ring methyl substitution the carcinogenicity of 3,8,13-trimethyl-TCQ was examined. This compound proved to be weakly active, skin tumours developing in 5 mice (9 per cent) during the period of skin painting and when the experiment was terminated after 18 months, the final tumour incidence was 17 per cent (carcinoma incidence 9 per cent, Table II).

Comparison of the potency of this compound with other TCQ derivatives is complicated because of its limited solubility (0·3 mg./ml. in benzene) and therefore the more soluble 3,8-dimethyl-TCQ derivative was also examined. Although

FIG. 2.—Development of skin tumours in mice treated with TCQ and methyl-substituted derivatives.

○———○ TCQ. ●———● 1-Methyl-TCQ.

■———■ 2-Methyl-TCQ. ×———× 3-Methyl-TCQ.

▲———▲ 4-Methyl-TCQ. □———□ 3,8,13-trimethyl-TCQ.

studies with this compound are not fully completed, the results indicate that it possesses only very weak carcinogenic activity (Table II). Thus during the period of skin painting tumours developed in 3 mice (9 per cent) but these regressed. Skin tumours arose in a further two mice after the completion of skin painting and at present (14 months) these are the only detectable tumours.

*The influence of halogen and hydroxyl substitution*

In order to assess the influence of peripheral ring blocking with substituents other than the methyl group, the halogen derivatives, 3-fluoro-TCQ and 3-bromo-TCQ were examined. The skin tumour incidence in mice treated with 3-fluoro-TCQ was 76 per cent (carcinoma incidence 61 per cent) which is comparable to that obtained with TCQ (Table II). However, as shown in Fig. 3, compared with TCQ, tumours developed much later in 3-fluoro-TCQ treated mice, the pattern of tumour induction being more comparable to that observed with 3-methyl-TCQ.

3-Bromo-TCQ also proved to be carcinogenic, skin tumours developing in 68 per cent of mice at risk (carcinoma incidence 68 per cent). The carcinogenicity of this compound is comparable to that of 3-fluoro-TCQ although, as shown in

Fig. 3, the latent period for tumour induction was less than that observed with either 3-fluoro- or 3-methyl-TCQ. Thus 50 per cent of mice at risk following treatment with 3-bromo-TCQ had developed skin tumours after 10 months, whereas with 3-fluoro- and 3-methyl-TCQ, 50 per cent tumour incidences were not observed until between 13 to 14 months.

FIG. 3.—Development of skin tumours in mice treated with halogen- and methoxyl-substituted derivatives.
○———○ TCQ. ●———● 3-Bromo-TCQ.
▲———▲ 3-Fluoro-TCQ □———□ 2-Methoxy-TCQ.

In view of the importance of the observation of low carcinogenic activity with 2-methyl-TCQ, the carcinogenicity of 2-methoxy-TCQ was determined to further assess the effect of substitution in the 2- position. This compound also proved to be weakly carcinogenic ; skin tumours developing in only 4 mice (12 per cent), and of these only 2 proved to be malignant (Table II).

*Histological findings*

The majority of skin tumours in mice treated with TCQ or peripheral ring substituted derivatives arose within the area of skin subjected to treatment. Additionally, a number of tumours arose on unpainted areas particularly around the head and shoulders, presumably due to the animals licking the compounds.

Histological examination of skin tumours indicated that the majority of malignant tumours were squamous cell carcinomata. In addition, there were also a number of basal cell carcinomas and an occasional sweat gland adenoma.

### DISCUSSION

The most important finding resulting from the investigation of carcinogenic activity in derivatives of tricycloquinazoline (Fig. 1) containing methyl substituents in the peripheral benzene rings is that 2-methyl-TCQ is virtually inactive whereas the other three possible monomethyl derivatives namely, 1-, 3- or 4-methyl-TCQ have comparable carcinogenic activities although somewhat less than that of the parent compound. That substitution in the 2-position produces complete or almost complete loss of carcinogenicity is further supported by the finding of low activity in 2-methoxy-TCQ (Table II); studies still incomplete with 2-hydroxy-TCQ (12 months) have failed to show any carcinogenic activity in this compound.

These findings suggest that the 2- position is involved in TCQ carcinogenesis. Since however, there are two other ring positions (7 and 12, see Fig. 1) which are equivalent to the 2-position in the TCQ molecule, it would be expected that blocking of only one position would reduce but not abolish carcinogenic activity. The reasons for this loss of carcinogenicity following methylation at the 2-position cannot yet be explained but one possible interpretation is that the primary action of the carcinogen at the tissue receptor involves at least a three-point union which is sterically influenced by substituents at the 2, 7 or 12 positions.

The mode of metabolism of tricycloquinazoline is still undetermined, but by virtue of the symmetry of the molecule, metabolic attack would be expected to occur at any of three sets of equivalent positions in the molecule, either in the peripheral benzene rings or at a C-N bond within the central nucleus. Substitution at the 2- position would not be expected to affect the metabolism of tricycloquinazoline and it has been shown (Baldwin, Palmer and Partridge, 1961) that the rates of metabolic breakdown of 2-methyl-TCQ and 3-methyl-TCQ are essentially similar. The finding that monomethyl substitution at the 2- position almost completely abolishes carcinogenic activity further indicates therefore that the whole TCQ molecule rather than a metabolite is the proximate carcinogen.

Derivatives containing methyl substituents at the 1-, 3-, or 4- positions are all carcinogenic although compared to the parent compound, activity is reduced. Introduction of additional methyl substituents causes a further decrease in carcinogenicity and thus 3, 8-dimethyl-TCQ and 3,8,13-trimethyl-TCQ are only weakly active (Table II). This gradual decrease in carcinogenic activity with the introduction of methyl substituents into the 3- or equivalent positions in the peripheral benzene rings (Fig. 1), it is suggested, may be due to an increasing interference with a carcinogen-receptor union.

The effects of methyl substitution on the carcinogenicity of tricycloquinazoline differ markedly from the findings with other epidermal hydrocarbon carcinogens (Badger, 1948, 1954). Thus in the 1,2-benzanthracene series, methyl substitution at any position in the anthracene residue generally enhances activity. Dimethylbenzanthracenes are also more potent than either of the monomethyl derivatives to which they are related. Monomethyl substitution in the angular benzene ring does not however enhance carcinogenicity and dimethyl derivatives in which one methyl substituent is in the angular ring are also inactive. The enhancement of carcinogenicity by methyl substitution in the 9- and 10- positions in benzanthracene has been interpreted in terms of electron densities at the K and L regions (Pullman and Pullman, 1955) although loss of activity by substitution in the angular ring has not been explained other than to suggest some possible stereochemical influence (Badger, 1954).

Enhancement of carcinogenic activity following methyl substitution has also been observed in the benzophenanthrene series. Comparison of the carcinogenicities of methylbenzopyrenes following subcutaneous injection has revealed that all excepting the 2′- and 3′- derivatives were active. Only one of these compounds (3′-methylbenzopyrene) has been examined following skin painting and in this case the compound was inactive (Schürch and Winterstein, 1935).

The most comprehensive investigation in recent years of the relationship of chemical structure to carcinogenic activity is that of Lacassagne *et al.* (1956) with the angular benzacridines and dibenzacridines. In these studies it was also shown that carcinogenicity was enhanced by methyl substitution ; the most important finding being that carcinogenic members were substituted in the meso-anthracenic carbon. Correlations with the electron densities of the K and L regions in these series of compounds have been employed in theoretical interpretations of their activities.

Hypotheses relating carcinogenic activity to electron densities of K and L regions appear to be irrelevant to TCQ since this compound contains no region identifiable as a K region. The evidence relating structure to activity in TCQ and its derivatives can be most logically accommodated in terms of the precision of stereochemical fit of the planar carcinogen by multiple bonding to a planar receptor site.

The almost complete suppression of activity by 2-methylation, 2-methoxylation, or 2-hydroxylation could be interpreted as evidence for the union of TCQ to a receptor at the 2-, 7- and 12- positions of the peripheral benzene rings. However a union specifically limited to these three positions would appear to require co-valent bonding of the carcinogen to the receptor, analogously to the co-valent binding of 1,2 : 5,6-dibenzanthracene to protein (Heidelberger, 1959). No evidence of such binding has been obtained (Baldwin, Palmer and Partridge, 1960) since TCQ can be removed from skin by simple washing with solvents ; evidently the carcinogen-receptor union involves weak intermolecular attractions.

Possible weak attractions are hydrogen bonding to the three peripheral nitrogen atoms, overlap of $\pi$-orbitals of the carcinogen and the tissue receptor, or multiple van der Waals bonding of a coplanar carcinogen and receptor. The amidine arrangement of the nitrogen atoms both in TCQ and in the planar bonded purine and pyrimidine pairs in DNA may be relevant to the possibility of the hydrogen bonding. Loss of activity induced by 2- substitution is readily envisaged as arising from a blocking of multiple weak bonding by steric hindrance

TABLE III.—*Correlation of the Skin Tumour Incidences of 3- or Equivalent Substituted TCQ Derivatives with the van der Waals radii of the Substituents*

| Substituent | van der Waals radius of substituent (Å) | Tumour incidence (percentage) |
|---|---|---|
| 3-H | 1·2 | 81 |
| 3-Fluoro | 1·35 | 76 |
| 3-Bromo | 1·95 | 68 |
| 3-Methyl | 2·0 | 59 |
| 3,8-Dimethyl | 2×2·0 | 8 |
| 3,8,13-Trimethyl | 3×2·0 | 17 |

between the substituent and any of three appropriately positioned functional groups adjacent to the receptor. Partial loss of activity caused by substituents in other positions in the peripheral homocyclic rings in TCQ could then be related to lower degrees of steric hindrance of the formation of a carcinogen-receptor union.

Evidence in harmony with the suggestion that stereochemical fit controls carcinogenic activity is implicit in the correlation between the incidence of tumours and the van der Waals radii of substituents of the 3- or equivalent 8- and 13-positions (Table III).

### SUMMARY

1.—Tricycloquinazoline (TCQ), a polyazapolycyclic hydrocarbon, is unrelated to any other epidermal hydrocarbon carcinogen and thus represents a new class of chemical compound for the investigation of the relationship between chemical structure and carcinogenicity.

2.—One of the four possible monomethyl derivatives, 2-methyl-TCQ, was virtually inactive following skin painting on mice, suggesting that the 2- position is involved in TCQ carcinogenesis. This is further supported by the finding of very low activity with 2-methoxy-TCQ.

3.—The three other possible monomethyl derivatives, 1-, 3- and 4-methyl-TCQ were all carcinogenic although their activities were less than that of the parent compound. Introduction of additional methyl substituents in positions equivalent to the 3- position caused further significant decreases in carcinogenic activity, and 3,8-dimethyl-TCQ and 3,8,13-trimethyl-TCQ were only weakly carcinogenic.

4.—The implications of these findings to TCQ carcinogenesis are discussed. The evidence relating structure to activity in TCQ and its derivatives can best be interpreted in terms of a stereochemical fit of the planar carcinogen by multiple bonding to a planar tissue receptor site. Unlike other polycyclic hydrocarbons, TCQ is not strongly bound to epidermal structures and thus the carcinogen-receptor union must involve weak intermolecular attractions.

Thanks are due to Mrs. M. Marshal and Miss R. Ellis for technical assistance. This work was supported by the Nottinghamshire Council of the British Empire Cancer Campaign.

### REFERENCES

BADGER, G. M.—(1948) *Brit. J. Cancer*, **2**, 309.—(1954) *Advanc. Cancer Res.*, **2**, 73.

BALDWIN, R. W., CUNNINGHAM, G. J. AND PARTRIDGE, M. W.—(1959) *Brit. J. Cancer*, **13**, 94.

*Idem*, PALMER, H. C. AND PARTRIDGE, M. W.—(1960) *Rep. Brit. Emp. Cancer Campgn.* **38**, 454.—(1961) *Ibid.*, **39**, 414.

HEIDELBERGER, C.—(1959) ‘ Ciba Foundation Symposium on Carcinogenesis ’. London (Churchill), p. 179.

Iball, J.—(1939) *Amer. J. Cancer*, **35**, 188.

LACASSAGNE, A., BUU-HOÏ, N. P., DAUDEL, R. AND ZAJDELA, F.—(1956) *Advanc. Cancer Res.*, **4**, 315.

PARTRIDGE, M. W., VIPOND, H. J. AND WAITE, J. A.—(1962) *J. chem. Soc.* In Press.

PULLMAN, A. AND PULLMAN, B.—(1955) *Advanc. Cancer Res.*, **3**, 117.

SCHÜRCH, O. and WINTERSTEIN, A.—(1935) *Z. physiol. Chem.*, **236**, 79.